# Hierarchical Attention Transformer-Based Sensor Anomaly Detection in Structural Health Monitoring

**DOI:** 10.3390/s25164959

**Published:** 2025-08-11

**Authors:** Dong Hu, Yizhou Lin, Shilong Li, Jing Wu, Hongwei Ma

**Affiliations:** 1 School of Environment and Civil Engineering, Dongguan University of Technology, Dongguan 523808, China; hudong_2000@163.com (D.H.); 18931110582@163.com (S.L.); tmahw@jnu.edu.cn (H.M.); 2 Guangdong Provincial Key Laboratory of Intelligent Disaster Prevention and Emergency Technologies for Urban Lifeline Engineering, Dongguan 523808, China; 3 School of Mechanical Engineering, Dongguan University of Technology, Dongguan 523808, China

**Keywords:** structural health monitoring, anomaly detection, long-span bridge, transformer, hierarchical attention

## Abstract

Structural health monitoring (SHM) is vital for ensuring structural integrity by continuously evaluating conditions through sensor data. However, sensor anomalies caused by external disturbances can severely compromise the effectiveness of SHM systems. Traditional anomaly detection methods face significant challenges due to reliance on large labeled datasets, difficulties in handling long-term dependencies, and issues stemming from class imbalance. To address these limitations, this study introduces a hierarchical attention Transformer (HAT)-based method specifically designed for sensor anomaly detection in SHM applications. HAT leverages hierarchical temporal modeling with local and global Transformer encoders to effectively capture complex, multi-scale anomaly patterns. Evaluated on a real-world dataset from a large cable-stayed bridge, HAT achieves superior accuracy (96.3%) and robustness even with limited labeled data (20%), significantly outperforming traditional models like CNN, LSTM, and RNN. Additionally, this study visualizes the convergence process of the model, demonstrating its fast convergence and strong generalization capabilities. Thus, the proposed HAT method provides a practical and effective solution for anomaly detection in complex SHM scenarios.

## 1. Introduction

Structural health monitoring (SHM) is an engineering management strategy that integrates sensor arrays, data collection, and analytical techniques to continuously or periodically evaluate the condition of civil, mechanical, and aerospace structures. Its goal is to maintain structural safety across the entire lifecycle or during critical periods. This approach deploys various sensor types throughout the structure: Fiber Bragg Grating sensors capture strain and temperature shifts, piezoelectric accelerometers record vibration signatures, acoustic emission sensors reveal the initiation and spread of material fissures via high-frequency elastic waves, and GPS units trace millimeter-scale displacements in large-scale structures such as bridges and high-rise buildings. All sensor data, conveyed through wired or wireless networks, is processed by a central system that applies signal analysis, pattern recognition, and damage diagnosis algorithms. By consolidating different data sources and employing intelligent evaluation methods, SHM provides early warnings of structural anomalies and accurately pinpoints damage locations. This ensures informed decision-making for maintenance and operation, safeguarding stable performance under complex service conditions [1,2,3,4,5].

In civil engineering, large structures like bridges, buildings, and dams are typically equipped with structural health monitoring systems as required by regulations. But in real-world conditions, sensor issues, diverse environmental factors, and dynamic vehicle loads can all produce unusual data. Such anomalies are common and may pose risks: if ignored, they could weaken the system’s ability to analyze and warn, and, in the worst case, they might hide real structural damage, creating safety gaps. Therefore, quickly identifying and removing these anomalies is essential for keeping SHM reliable. It is important to note that the anomalies discussed here mainly refer to anomalies caused by outside influences, unlike those arising from the structure’s own damage or deterioration [6,7,8,9].

Conventional anomaly detection methods for this task are often divided into three main categories: statistical, distance-based, and model-based approaches. Statistical methods generally assume that the data follow a specific distribution and then detect anomalies by measuring how far observed values stray from that distribution. For example, Edgeworth et al. [10] first proposed identifying outliers by how much they diverge from the normal distribution, and Anscombe et al. [11] later refined these ideas using standard deviations or quartile ranges. Although these methods are relatively straightforward, they tend to underperform with data that are non-normal, high-dimensional, or non-stationary, and they usually depend on delicate parameter choices. In contrast, distance-based methods center on local neighborhood density and use an anomaly score based on the distance between a data point and its neighbors or the density gap in that region. A notable example is the Local Outlier Factor, introduced by Knorr et al. [12], which flags outliers in sparse regions using a distance threshold. However, these methods often struggle in higher dimensions due to challenges arising from large datasets and their sensitivity to parameter settings. Model-based approaches place more focus on describing the processes or behaviors within a system, typically by building a reference model of normal conditions and identifying any deviations. For example, Goebel et al. [13] introduced a data-fusion and self-learning technique for sensor-failure detection that enables indirect correction of anomalous readings, while Lo et al. [14] developed distributed diagnostic tools for nonlinear wireless sensor networks, strengthening system independence and resilience. Additionally, Huang et al. [15] combined statistical hypothesis testing with principal component analysis (PCA) in a hybrid diagnostic framework to detect drift- or offset-type anomalies, improving fault detection and tolerance. Though these methods have advanced both in theoretical research and in small-scale engineering practice, they still face hurdles when confronted with the increasingly high-dimensional, highly nonlinear, and long-duration data found in structural health monitoring (SHM). Their adaptability, feature-extraction capabilities, and overall robustness often fall short of meeting the stringent accuracy and low-false-alarm requirements of complex, real-world situations.

In recent years, deep learning methods have become popular in sensor anomaly detection because they can automatically capture important features from large datasets [16]. Convolutional neural networks (CNNs) are a good example of this. Lin et al. [17] developed a CNN-based method to illustrate how features evolve at different network depths: shallow layers typically detect basic broadband filtering features, middle layers focus on capturing multi-frequency modal characteristics, and deep layers can naturally identify structural vibration modes from raw signals. Following this concept, Tang et al. [18] developed a method using two-channel inputs combining time-domain and frequency-domain representations, significantly enhancing the model’s detection accuracy for different types of anomalies and its ability to generalize. Zhang et al. [19] further improved detection with imbalanced datasets by applying data augmentation along with one-dimensional CNNs, achieving stable results when tested on a large bridge monitoring dataset containing over 20,000 samples collected continuously for a month, thus showing the importance of dataset size on model performance. Recurrent neural networks, especially those using LSTM, have also been widely adopted because they handle time-dependent relationships effectively. Son et al. [20] applied an LSTM encoder–decoder to successfully distinguish anomalies caused by structural damage from those related to sensor faults. Zhang et al. [21] further improved anomaly recognition in real time by introducing a dual-threshold method that helps reduce false alarms. Generative adversarial networks (GANs) have shown potential as well, training a generator and discriminator in competition to closely match the distribution of normal data without relying on labeled examples. Deng et al. [22] proposed a frequency-domain method for filtering out seemingly normal signals, while Tu et al. [23] introduced a weighted GAN (CWGAN), based on maximum correntropy, to dynamically adjust sample weights, enhancing the model’s robustness against non-Gaussian noise. Mao et al. [24] combined GANs with autoencoders, integrating the strengths of unsupervised training and improved pattern recognition capabilities for irregularities. Lin et al. [25] proposed a damage detection method based on variational autoencoders, which leverages feature correlation from limited sensor data to identify structural damage accurately without requiring baseline information. Wang et al. [26] proposed a novel online meta-learning framework for sensor fault diagnosis using limited data. Their method integrates a 1D CNN with model-agnostic meta-learning to rapidly adapt to new fault scenarios, followed by a dual Kalman filter for fault severity estimation and state tracking. Similarly, Civera et al. [27] developed an unsupervised DBSCAN-based operational modal analysis method that enables automatic extraction of modal parameters under ambient excitations, providing a robust baseline-free tool for long-term bridge monitoring.

Despite these advances, three main issues still remain in practical SHM scenarios. First, current methods depend heavily on large labeled datasets, limiting their use in situations where resources or labeled samples are scarce. Second, traditional recurrent models like RNNs and LSTMs often face problems such as vanishing gradients, causing difficulty in effectively learning long-term dependencies and identifying anomalies across long sequences. Third, sensor data typically suffer from significant class imbalance, which reduces the stability of models and makes generalization to new data more challenging [28,29].

Recently, more advanced Transformer-based models have started showing better performance in various tasks because their self-attention mechanism can naturally handle long-range dependencies, making them particularly suitable for time-series sensor data spanning extended periods. Initially proposed by Vaswani et al. [30] and later enhanced through models like BERT and GPT, Transformers have become widely used in NLP, and, more recently, they are increasingly applied in time-series prediction tasks, such as financial forecasting and power load analysis, due to their strong modeling capabilities over long sequences and parallel processing strengths [31,32]. However, traditional Transformers face issues of quadratic computational complexity, making it challenging to handle extremely long sequences typical in large-scale SHM applications. To overcome this, researchers have proposed hierarchical Transformers, such as HitAnomaly [33] and HTS-AT [34], which divide lengthy sequences into smaller segments using layered architectures, capturing detailed local patterns while maintaining global context. These methods have effectively enhanced anomaly detection accuracy for various data sources like system logs and audio signals.

Motivated by these advancements, this study introduces the hierarchical attention Transformer (HAT) into SHM sensor anomaly detection, aiming to tackle two critical challenges in real-world applications: dependency on large labeled datasets and difficulty in modeling long sequences. By modifying the hierarchical structure specifically for SHM data, HAT reduces computational demands when handling long-duration sequences and improves the detection of anomalies spanning different temporal scales, making it particularly effective in scenarios with limited annotations and complex time-series inputs. Its core contributions are as follows: First, we customize a hierarchical attention-based architecture for SHM sensor anomaly detection by adapting the HAT framework to effectively capture both local (intra-segment) and global (inter-segment) temporal dependencies. To the best of our knowledge, this is the first study to introduce a HAT-based modeling strategy into the domain of SHM anomaly detection. This adaptation enhances the model’s ability to represent multi-scale anomaly patterns commonly observed in SHM data and reduces computational overhead when processing long-duration sequences. Second, extensive experiments on a month-long real-world bridge monitoring dataset (26,448 samples) demonstrate that our method achieves a high detection accuracy of 96.3% using only 20% of labeled data. This result underscores the model’s strong potential for practical SHM applications, particularly in scenarios with limited annotations and pronounced class imbalance. The framework not only generalizes well under weak supervision but also converges rapidly during training, offering a competitive balance between effectiveness and efficiency. Third, the model supports end-to-end learning from raw time-series inputs and enables anomaly visualization through progressive feature separation. It eliminates the need for manual feature engineering while producing interpretable feature representations, thus improving both usability and applicability in complex and evolving SHM environments.

The remainder of this paper is organized as follows. Section 2 introduces the proposed methodology, detailing the core components of the model including multi-head attention, positional encoding, and hierarchical feature extraction, followed by the evaluation metrics used in this study. Section 3 presents the experimental results, beginning with a description of the dataset and continuing with data preprocessing procedures. This is followed by an in-depth performance evaluation, including quantitative comparisons with baseline models, analysis of classification results for each anomaly type, and comparisons with results reported in representative SHM studies. The section also includes a visual analysis of the model’s convergence process during training. Finally, Section 4 summarizes the key findings and discusses the implications and future directions of this work.

## 2. Methodology

### 2.1. Overview of the Approach

**Figure 1:**
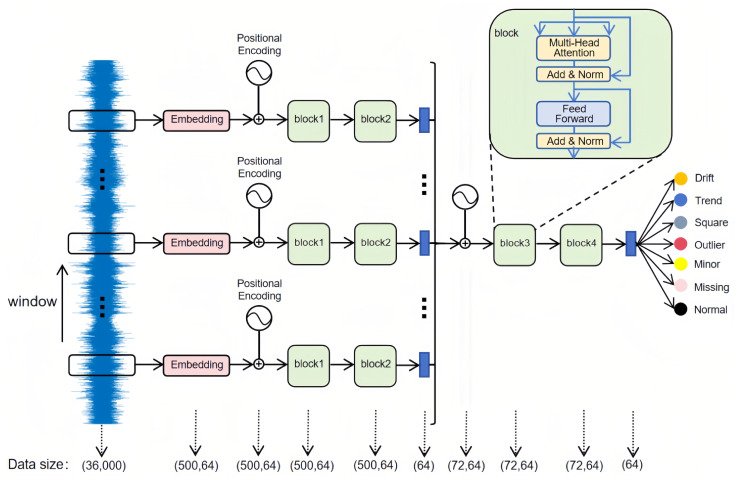
Hierarchical attention transformer architecture.

This study proposes a hierarchical attention Transformer-based approach for anomaly detection in SHM, comprising four main modules: an input partition and embedding module, an intra-segment Transformer encoder, an inter-segment Transformer encoder, and a linear layer. Figure 1 illustrates the overall workflow.

The raw time-series signal has a length of 36,000 and a sliding window that advances by 500 time steps splits this sequence into 72 non-overlapping segments of 500 time steps each. Each part is then passed through an embedding layer for linear transformation, producing a (500, 64) vector representation, and augmented with sine–cosine positional encodings to preserve temporal order. Subsequently, the segment passes through two Transformer encoder layers (block 1 and block 2), which capture local temporal dependencies; average pooling then condenses the encoded features into a 64-dimensional representation. All 72 compressed segment features are concatenated into a (72, 64) sequence for further processing by another two-layer Transformer encoder (block 3 and block 4), thereby modeling extended dependencies across different time segments; here, positional encodings again maintain the sequential structure among segments. Finally, the model applies average pooling to compress the output into a single 64-dimensional vector and uses a linear layer to classify it into one of seven categories: Normal, Missing, Minor, Outlier, Square, Trend, or Drift. By using this multi-layered approach, the model captures both local and global information within long sensor data, effectively identifying complex anomaly patterns common in structural health monitoring.

To explain how the method works internally, we focus on three main components: multi-head attention, positional encoding, and hierarchical feature extraction. These modules work closely together, forming a structured temporal modeling framework. This combination helps the model achieve quick convergence even with limited labeled data while effectively modeling long-term dependencies. By carefully examining the purpose and functionality of each component, we better understand how they contribute to enhancing time-domain representations and improving anomaly detection accuracy and robustness in practical engineering tasks.

#### 2.1.1. Multi-Head Attention

This model achieves fast convergence even with limited labeled data, mainly due to the multi-head attention mechanism included in each block. The term “multi-head” refers to dividing the input feature vectors into multiple smaller heads, where attention computations are performed separately within each group. After these independent computations, the results are combined into one output. This allows the model to identify a broader set of features and interactions from different angles, enhancing the extraction of useful information from sequences. Specifically, single-head attention is computed following Equation (1), where Q, K, and V represent Query, Key, and Value, respectively, and $d_{k}$ denotes the dimension of the Key vectors.

(1) $$\mathrm{Attention}\left(Q,K,V\right)=\mathrm{softmax}\left(\frac{QK^{⊤}}{\sqrt{d_{k}}}\right)V$$

Multi-head attention follows the computations shown in Equations (2) and (3), where $W_{i}^{Q}$, $W_{i}^{K}$, and $W_{i}^{V}$ are trainable projection matrices corresponding to the *i*-th attention head. These projection matrices map the input queries (Q), keys (K), and values (V) to different subspaces for each head, allowing the model to focus on different aspects of the input sequence. Each head captures distinct features within a lower-dimensional subspace, providing the model with multiple perspectives on the data. In Equation (3), each attention head, ${\mathrm{head}}_{i}$, computes attention scores by performing a scaled dot-product attention between the projected queries, keys, and values, which assigns different weights to the input tokens based on their relevance to each query. The outputs from each head are concatenated in Equation (2), and the final output is computed by multiplying the concatenated attention heads with the output projection matrix $W^{O}$. This process allows the model to integrate the diverse perspectives learned by each head into a richer, more comprehensive representation, capturing a wider range of relationships and hidden details in the input data.

(2) $$\mathrm{MultiHead}\left(Q,K,V\right)=\mathrm{Concat}\left({\mathrm{head}}_{1},{\mathrm{head}}_{2},\ldots ,{\mathrm{head}}_{h}\right)W^{O}$$

(3) $${\mathrm{head}}_{i}=\mathrm{Attention}\left(QW_{i}^{Q},KW_{i}^{K},VW_{i}^{V}\right)$$

#### 2.1.2. Positional Encoding

Time-based dependence is a crucial feature in time-series analysis as each point’s position within the sequence influences the overall structure of the data. In a standard Transformer model, sequence elements are processed in parallel, meaning the model lacks an inherent way to capture the order or relative positions of elements in the sequence. To address this, positional encodings are introduced. These encodings are added to the input vectors to encode the position of each time step in a unique manner. Specifically, sine and cosine functions, as defined in Equations (4) and (5), are used to generate the positional encodings. The idea behind this method is that each time step is assigned a set of trigonometric values, with different frequencies, that reflects its relative position in the sequence. The positional encoding for each position, pos, and dimension, *i*, is calculated using these sine and cosine functions, ensuring that each time step receives a unique representation based on its position. This encoding allows the Transformer to incorporate time-order information and use it to better model sequential dependencies.

(4) $$\mathrm{PE}\left(pos,2i\right)=\sin\left(\frac{pos}{10000^{\frac{2i}{d_{\mathrm{model}}}}}\right)$$

(5) $$\mathrm{PE}\left(pos,2i+1\right)=\cos\left(\frac{pos}{10000^{\frac{2i}{d_{\mathrm{model}}}}}\right)$$

In these equations, PE(pos,2i) and PE(pos,2i+1) correspond to the positional encodings for the 2i-th and 2i+1-th dimensions of the encoding, respectively. The function $10,000^{\frac{2i}{d_{\mathrm{model}}}}$ introduces different frequencies for each dimension, ensuring that each position is represented with distinct periodic values. These encodings are added directly to the input embeddings, providing the model with information about the relative positions of elements in the sequence, allowing it to learn time-dependent relationships more effectively.

#### 2.1.3. Hierarchical Feature Extraction

In this work, such position encoding is applied at both the intra-segment level and the subsequent inter-segment stage to capture multiscale dynamics in extra-long SHM time-series data. First, the original signal is split into fixed-length segments, each representing a short window of structural responses. A local Transformer encoder then models the temporal dependencies within each segment, thereby highlighting subtle local fluctuations. Afterward, these segment-wise features are consolidated into a higher-dimensional sequence, which is fed into a global Transformer encoder for inter-segment attention. This hierarchical approach reduces the computational complexity typical of standard Transformers, which scales as $O(n^{2})$, by breaking long sequences into smaller segments while still preserving overall context.

Additionally, it allows the model to perceive fine-grained details alongside larger patterns in the data, effectively building up information from local segments to global contexts. Because subtle anomalies can sometimes be masked by dominant trends, using two-level attention helps reveal less noticeable pseudo-anomalies and overlapping irregularities across multiple temporal scales. Thus, the design increases sensitivity to true anomalies and reduces false alarms, making the approach particularly suitable for practical structural health monitoring tasks.

### 2.2. Evaluation Metrics

To comprehensively gauge the model’s anomaly detection performance for structural health monitoring, this study employs four established classification metrics: Accuracy, Precision, Recall, and F1-score and further uses a Confusion Matrix to visualize prediction outcomes across different categories. These evaluation methods characterize the model’s performance from multiple perspectives.

Precision, defined by Equation (6), focuses on how many of the samples predicted as anomalies are truly anomalous, which is particularly vital when false alarms are costly or unacceptable in practice. Recall, calculated according to Equation (7), measures the model’s ability to capture genuine anomalies and thus indicates its effectiveness in minimizing overlooked outliers. Accuracy, as shown in Equation (8), reflects the proportion of correctly classified samples across all categories and represents the model’s overall recognition capability. Finally, the F1-score, defined in Equation (9) as the harmonic mean of Precision and Recall, provides a more balanced evaluation in scenarios where these two metrics often trade off [35,36].

(6) $$\mathrm{Precision}=\frac{TP}{TP+FP}$$

(7) $$\mathrm{Recall}=\frac{TP}{TP+FN}$$

(8) $$\mathrm{Accuracy}=\frac{TP+TN}{TP+TN+FP+FN}$$

(9) $$F1\text{-}\mathrm{score}=2\times \frac{\mathrm{Precision}\times \mathrm{Recall}}{\mathrm{Precision}+\mathrm{Recall}}\times 100$$

## 3. Results

### 3.1. Dataset

**Figure 2:**
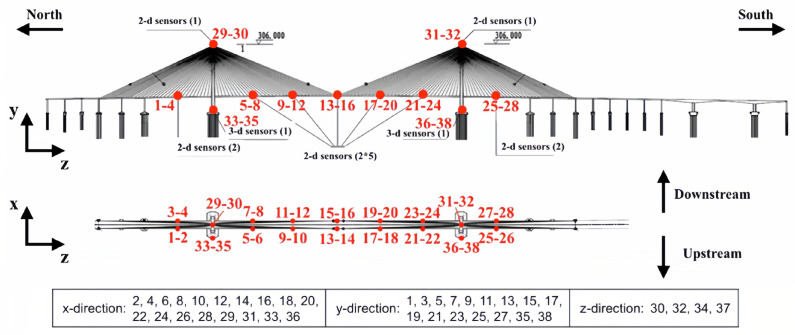
Sensor locations on the long-span cable-stayed bridge.

As illustrated in Figure 2, the acceleration dataset utilized in this study originates from a real-world long-span cable-stayed bridge located in China, featuring a main span of 1088 m, two side spans of 300 m each, and two bridge towers, each rising to 306 m [37]. A total of eighteen accelerometers, providing 38 measurement channels, were strategically deployed across the bridge: fourteen dual-channel devices along the deck, two dual-channel devices at the tower tops, and two tri-channel units at the tower bases. All sensors operated at a sampling rate of 20 Hz. The dataset captures structural behavior over a two-month monitoring period; specifically, 5654 samples, representing 20% of the first month’s records, are employed for training, whereas 26,448 samples from the subsequent month constitute the test set. This dataset is sourced from the 1st International Project Competition for Structural Health Monitoring (IPC-SHM 2020).

**Figure 3:**
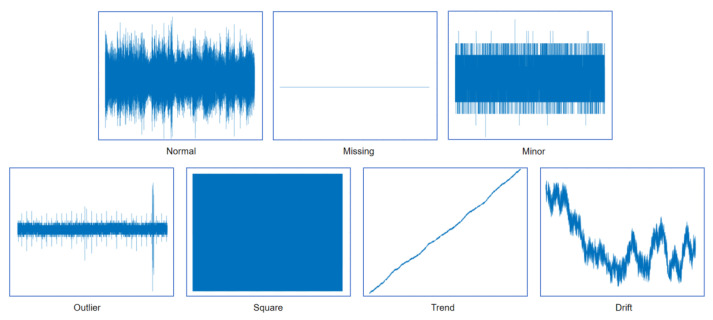
Types of data anomalies.

Table 1 summarizes the distribution and characteristics of normal data and six defined anomaly categories within the one-month dataset, while Figure 3 provides representative examples for each data pattern.

### 3.2. Experiments

To rigorously assess the performance of the proposed model under varying levels of supervision, this study designs three experimental conditions, referred to as Case 1, Case 2, and Case 3, corresponding to training label proportions of 20%, 50%, and 100%, respectively. This setup reflects practical challenges commonly encountered in structural health monitoring, where fully annotated datasets are often unavailable due to the substantial cost of manual labeling. Among these conditions, Case 1 represents the most constrained supervision scenario and serves as the primary basis for model comparison, highlighting the model’s effectiveness under limited label availability. To preserve the original class distribution and ensure a balanced representation of each anomaly category, 20% of the samples from each class are randomly selected to form the low-label dataset. Based on this, the dataset is then split, with the first 80% of the selected samples used for training and the remaining 20% used for validation. Additionally, downsampling and normalization are applied to reduce computational complexity and improve numerical stability during training. The experimental environment, including hardware configuration and software frameworks, is described to ensure the reproducibility and fairness of the evaluation.

#### 3.2.1. Random Sampling of 20% Data

**Figure 4:**
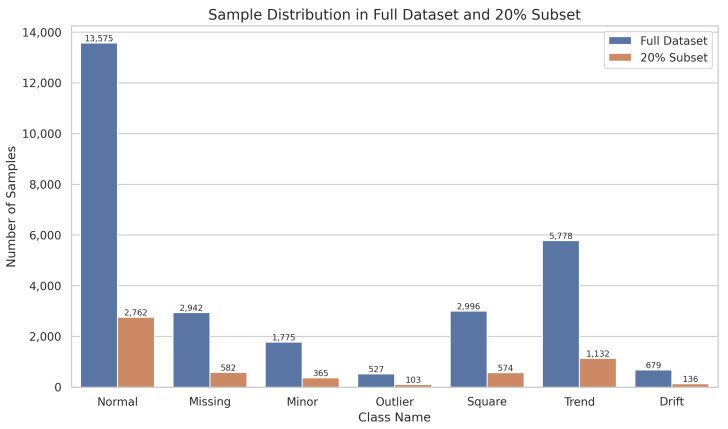
Sample distribution across different anomaly categories.

A random selection of 20% of each category from one month’s data, as summarized in Table 2, confirms that the distribution of sampled data remains consistent with the original set, albeit imbalanced, which both raises classification complexity and aligns with real-world engineering scenarios. The corresponding sample distribution is visualized in Figure 4, clearly illustrating the proportional relationships across different anomaly types.

#### 3.2.2. Downsampling and Normalization

**Figure 5:**
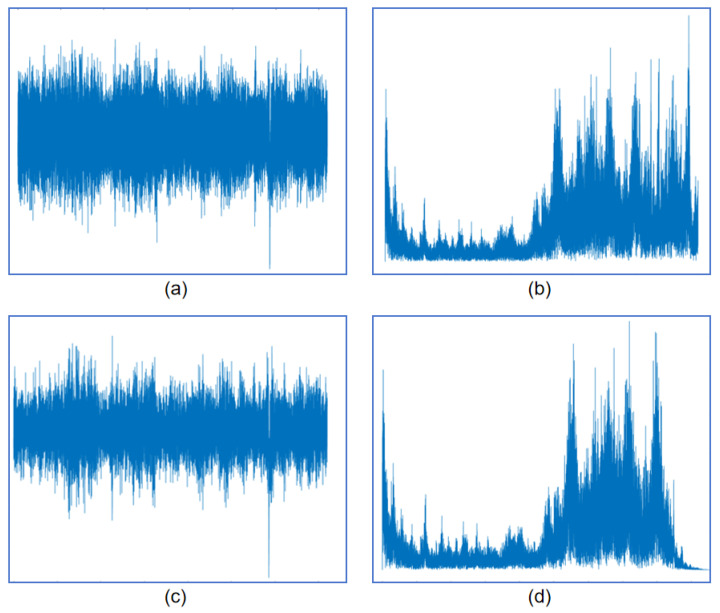
Comparison of the original and downsampled signals in time and frequency domains: (**a**) time-domain signal at 20 Hz; (**b**) frequency-domain signal at 20 Hz; (**c**) time-domain signal downsampled to 10 Hz; (**d**) frequency-domain signal after downsampling to 10 Hz.

To lessen computational demand, expedite detection, and improve efficiency, the original 20 Hz sampling rate is first subjected to low-pass filtering with a cutoff frequency of 5 Hz. According to the Nyquist Theorem, this process ensures that the new sampling rate remains sufficiently high, being twice the maximum frequency, thereby preventing aliasing artifacts by discarding signal components above 5 Hz. Subsequently, the data are downsampled to 10 Hz. After downsampling, Z-score normalization is applied, standardizing the data with a mean of zero and a variance of one. This stabilization accelerates model convergence and keeps gradients within a reasonable range during training. As visualized in Figure 5, the time-domain and frequency-domain signals of the original 20 Hz data, along with their downsampled and normalized counterparts, are compared. It can be observed that no aliasing occurs after downsampling, essential information is preserved, and high-frequency noise is effectively filtered out, thus reducing the interference to the model input and enhancing the overall computational efficiency.

#### 3.2.3. Experimental Environment

All experiments were conducted on a high-performance setup summarized in Table 3, including an Intel Xeon Gold 6430 CPU and an NVIDIA GeForce RTX 4090 GPU with 24 GB memory. The environment was based on Windows 11, Python 3.8, PyTorch 2.0.0, and CUDA 11.8, ensuring efficient processing of the large SHM dataset.

HAT is used with the key training parameters settings summarized in Table 4 being determined using the validation set for hyperparameter tuning. Numerous configurations were tested, and the final settings were chosen as they consistently yielded the best performance on the validation set in terms of both training stability and detection accuracy. A learning rate of 0.0001 and a dropout rate of 0.1 were selected to stabilize training and prevent overfitting. The model used Adam optimizer and CrossEntropyLoss, with a hidden dimension of 64, a feedforward size of 256, and four attention heads. These settings helped balance model complexity and detection performance.

The LSTM, RNN, and 1D-CNN models used in this study each consist of three layers with 128 hidden units (for LSTM and RNN) or three convolutional layers (for 1D-CNN) with 32, 64, and 128 filters, respectively. All models use ReLU activation and dropout (0.15 for LSTM, 0.2 for RNN, and 0.18 for 1D-CNN). The Adam optimizer was used with learning rates of 0.001 for LSTM and 1D-CNN, and 0.002 for RNN, and cross-entropy loss was used for training. Hyperparameters were tuned based on validation accuracy, and early stopping was applied to avoid overfitting.

### 3.3. Result Analysis

**Figure 6:**
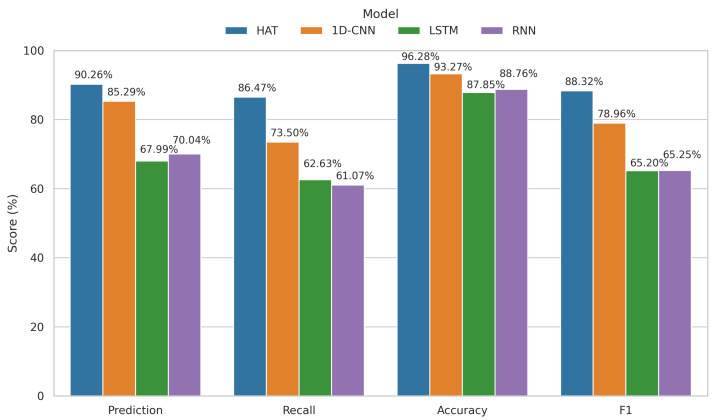
Performance comparison of different models (HAT, 1D-CNN, LSTM, and RNN) on key metrics (Prediction, Recall, Accuracy, and F1-score) under 20% labeled data (Case 1).

To comprehensively evaluate the proposed hierarchical attention Transformer (HAT) for sensor anomaly detection, three widely used baseline models, namely, 1D-CNN, LSTM, and RNN, are selected for comparison. All models are trained using the same dataset and preprocessing pipeline, with their optimal hyperparameters. As summarized in Table 5 and visualized in Figure 6, the HAT approach achieves superior performance across four metrics: Prediction Accuracy, Recall, overall Accuracy, and F1-score, under different labeled data proportions including 20%, 50%, and 100%.

Under the most challenging low-label condition where only 20% of data are annotated, HAT reaches a prediction accuracy of 90.26%, outperforming 1D-CNN (85.29%), LSTM (67.99%), and RNN (70.04%). Its Recall also leads at 86.47%, while 1D-CNN, LSTM, and RNN reach 73.50%, 62.63%, and 61.07%, respectively. In terms of the F1-score, HAT attains 88.32%, significantly higher than 78.96% for 1D-CNN, and over 20 percentage points higher than LSTM and RNN. As more labeled data become available, the performance of all models improves; however, HAT consistently maintains the leading position across all scenarios. In the fully labeled setting, HAT’s F1-score rises to 90.14%, substantially outperforming the baselines, which demonstrates its strong generalization and robustness for practical anomaly detection in structural health monitoring tasks.

In Table 6, we compare the detection performance of HAT under Case 1 conditions against two representative existing models—those proposed by Bao et al. and Tang et al. HAT outperforms both in every metric, reaching a prediction accuracy of 90.26%, recall of 86.47%, overall accuracy of 96.28%, and an F1-score of 88.32%. Relative to Bao et al.’s method, these figures represent improvements of 11.1%, 5.76%, 9.28%, and 8.39%, respectively, while compared to Tang et al.’s approach, HAT gains 1.07%, 7.00%, 2.08%, and 4.27%, underscoring its effectiveness when dealing with real-world engineering data, limited labels, and complex anomaly patterns.

**Figure 7:**
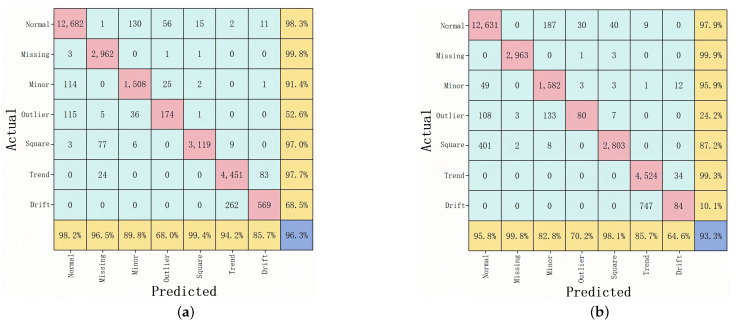
(**a**) Confusion matrix for HAT model; (**b**) confusion matrix for 1D-CNN.

**Figure 8:**
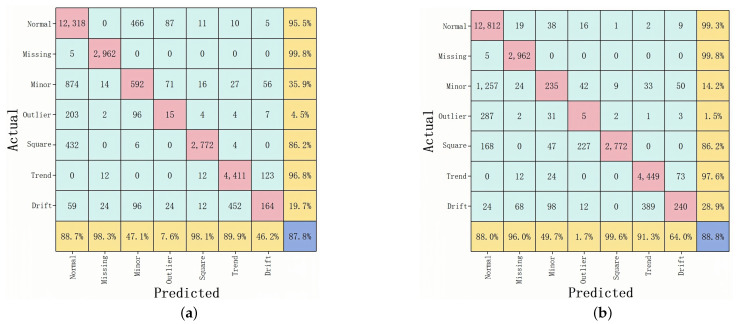
(**a**) Confusion matrix for LSTM; (**b**) confusion matrix for RNN.

As shown in Figure 7 and Figure 8, a deeper look at the confusion matrices clarifies how each model performs on specific anomaly categories. Each matrix shows the model’s ability to classify anomalies in the case where 20% of the data are labeled. From the HAT confusion matrix, it is clear that the model achieves high recall and prediction accuracy across all seven types; in particular, Normal, Missing, Minor, Square, and Trend classes show recall values of 98.3%, 99.8%, 91.4%, 97.0%, and 97.7%, respectively. Although Outlier and Drift are relatively difficult to detect and account for a smaller portion of the dataset, HAT still manages recall rates of 52.6% and 68.5% for these classes, though they do modestly lower its overall F1-score. By contrast, 1D-CNN performs well for Normal (97.9%), Missing (99.9%), Minor (95.9%), and Trend (99.3%) but plummets for Outlier (24.2%) and Drift (10.1%), revealing a lack of sensitivity to isolated outliers and gradual drift anomalies. The root cause is likely the CNN’s strong focus on local features, which makes it difficult to capture extended temporal patterns. Although LSTM preserves good recall for Normal, Missing, and Trend—95.5%, 99.8%, and 96.8%, respectively—its performance for Minor, Outlier, Square, and Drift drops, substantially, to 35.9%, 4.5%, 86.2%, and 19.7%. This decline is partly due to long-sequence gating constraints that weaken LSTM’s ability to handle imbalanced data and far-reaching dependencies. RNN experiences similar but more volatile results; it manages a recall of just 14.2% for Minor, 1.5% for Outlier, and 28.9% for Drift—lower overall than HAT’s 68.5% for Drift—highlighting serious memory decay issues with long inputs, especially when anomalies evolve gradually or span multiple cycles.

HAT demonstrates a more balanced and consistent detection capability across all anomaly types. Even under challenging conditions such as ambiguous boundaries between categories and limited labeled data, it maintains strong recall, prediction accuracy, and F1-scores. This performance advantage underscores the strengths of its hierarchical, multi-scale design for dealing with real-world sensor data in a weakly supervised environment.

**Figure 9:**
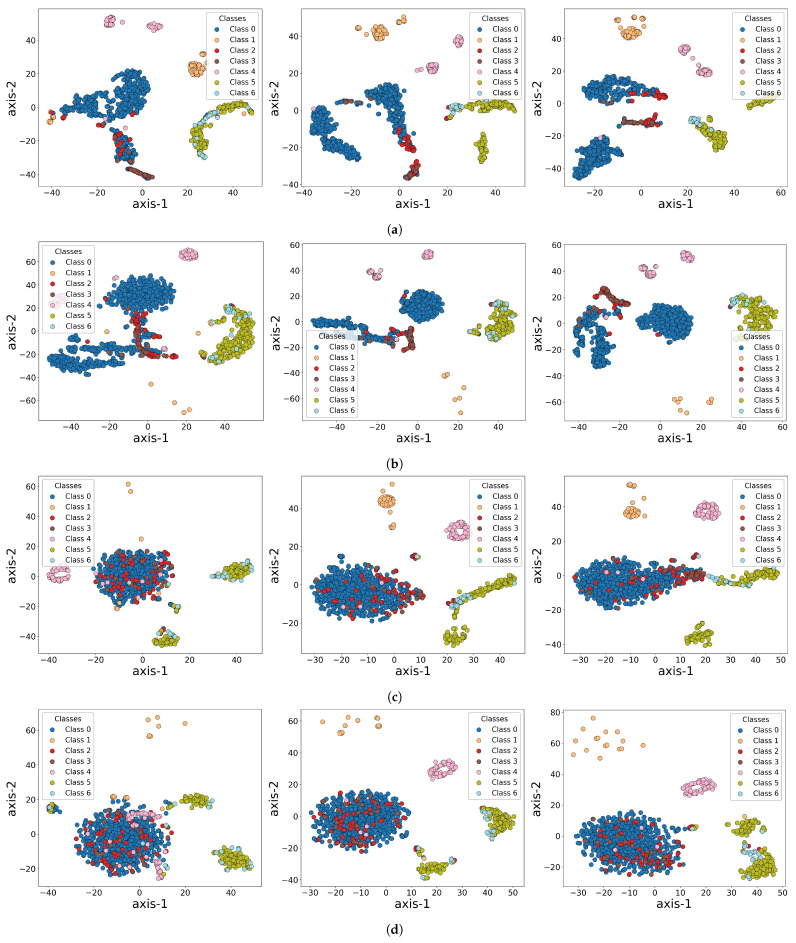
Visualization of feature representations extracted from HAT, 1D-CNN, LSTM, and RNN models at different training stages (Epoch 1, Epoch 15, and Epoch 25). (**a**) HAT; (**b**) 1D-CNN; (**c**) LSTM; (**d**) RNN. These visualizations demonstrate how the feature spaces evolve during training across different models.

To further explore how the models learn features and differentiate categories during training, we visualized the feature representations extracted by HAT, 1D-CNN, LSTM, and RNN at different training epochs (Epoch 1, 15, and 25), as shown in Figure 9. Using t-SNE, we projected high-dimensional feature vectors into a two-dimensional plane, with seven classes (Class 0 to Class 6) indicated by distinct colors. Each row in the figure represents one model’s embedding space at different epochs, enabling a comparison of how their discriminative abilities evolve. HAT quickly establishes clear clusters, with certain classes like Class 0, Class 4, and Class 5 forming stable regions early in training. By Epoch 25, decision boundaries become highly distinct, indicating HAT’s rapid convergence and efficient feature extraction. In contrast, 1D-CNN initially shows significant overlap, with some classes beginning to separate by Epoch 15, but it takes until Epoch 25 to develop a more noticeable cluster structure. LSTM evolves more slowly, with heavy overlap at Epoch 1 and only a few classes starting to cluster by Epoch 25. RNN shows a similar slow convergence, with incomplete clustering throughout the first 25 epochs, consistent with its lower performance metrics compared to HAT. These observations reflect that LSTM and RNN have more difficulty distinguishing complex anomaly patterns.

**Figure 10:**
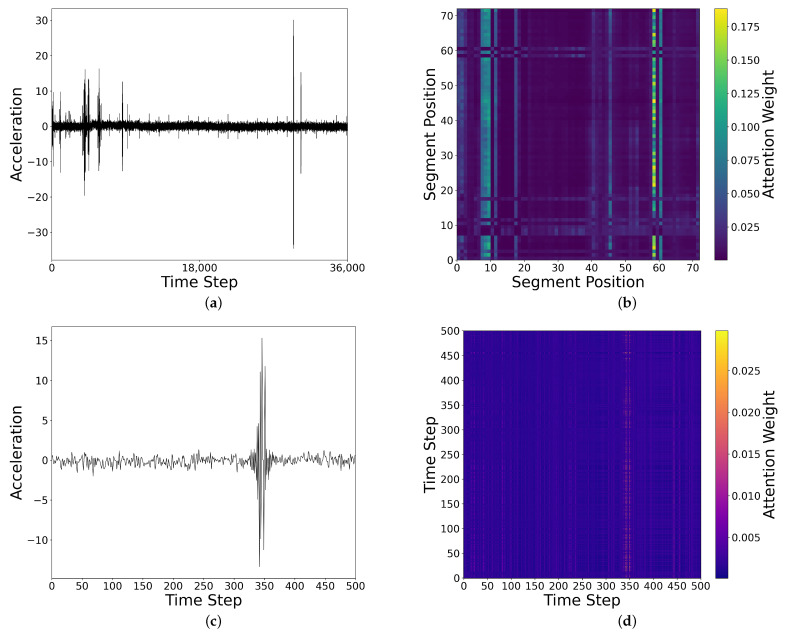
Visualization of inter- and intra-segment attention for an outlier sample: (**a**) sample time series; (**b**) inter-segment attention map; (**c**) time series of one segment; (**d**) intra-segment attention map.

**Figure 11:**
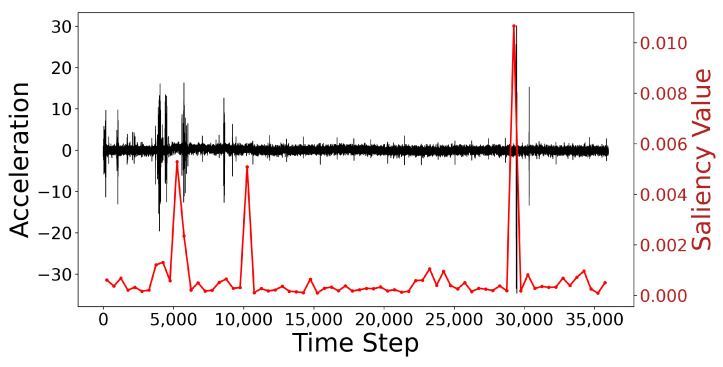
Visualization of time series and saliency map.

To enhance the interpretability of the model, we present the visualizations of the attention maps and saliency maps in Figure 10 and Figure 11, aiming to clarify the key features the model learns from the sensor signals. Figure 10 shows the visualizations of both inter- and intra-segment attention. Figure 10a presents the raw time-series data, while Figure 10b illustrates the inter-segment attention map. In the global attention map, we observe significant attention enhancement at the anomaly points (such as the peak locations), especially around segment 60, indicating that the model effectively focuses on these abnormal patterns. Figure 10c shows the time series for one of the segments, while Figure 10d displays the intra-segment attention map for this segment. The intra-segment attention map reveals that the model also pays high attention to the peak locations, suggesting that the model can effectively capture significant changes within the local context of the signal. It is important to note that all the attention maps are calculated by averaging the values from the four attention heads, which helps balance the contributions from different heads and further enhances the model’s stability and expressiveness. Through these visualizations, we gain a more intuitive understanding of how the model processes sensor data and focuses on the critical anomalous regions.

Additionally, Figure 11 shows the time-series data and their corresponding saliency map. The black curve represents the original acceleration time-series data, while the red curve shows the variation in saliency values. Notably, at the anomaly point near time step 30,000, there is a clear peak in the saliency values, indicating the model’s heightened attention at these locations. These anomaly points align with the time-series data that the model focuses on, further validating the model’s sensitivity in anomaly detection. The saliency map is generated using the Integrated Gradients (IG) method, reflecting the influence of each time step on the model’s decision-making process. This visualization allows for a more intuitive understanding of how the model makes classification decisions based on specific patterns in the time-series data.

As shown in Table 7, the resource demands and inference times for the models are presented. Due to the larger number of parameters and the computational nature of the attention mechanism in Transformer-based models, the HAT model exhibits a longer inference time. However, this is acceptable given that the model provides the best accuracy, and the inference time of approximately 0.8 s for processing one hour of sensor data meets real-time requirements for practical engineering applications.

From the perspective of explainability, this visualization provides a concrete “de-black-boxing” perspective on HAT: instead of being an inscrutable deep network, HAT demonstrates how it progressively learns and extracts features tightly linked to different anomalies. The movement of classes from an intermixed state to distinct clusters highlights the effectiveness of HAT’s multi-head self-attention and hierarchical modeling strategy, including the role of positional encoding. In particular, the dual modeling of local and global time scales ensures that both minor, localized fluctuations and broader, long-term trends are assimilated, giving HAT a feature space with a high degree of separability. This grouping is not only statistically meaningful but also structurally interpretable in light of the model’s architecture.

## 4. Discussion and Conclusions

In this study, we address two fundamental challenges in SHM: dependency on large labeled datasets and difficulty in modeling long sequences. To address the former, our method achieves strong detection performance with only 20% labeled data, demonstrating its effectiveness in low-label settings. To overcome the latter, our method utilizes a hierarchical feature extraction structure, which captures both local and global temporal patterns from raw SHM signals, significantly improving anomaly detection accuracy. Experimental validation on a month-long real-world bridge monitoring dataset demonstrates the robustness and efficiency of the proposed model, highlighting its potential for practical deployment in complex SHM environments.

The key findings of this work can be summarized as follows:

1. HAT represents a potent new paradigm for sensor anomaly detection: It significantly outperformed baseline architectures and opens a promising research avenue for scalable monitoring of complex infrastructures. Its segment-wise hierarchical attention mirrors the inherent multiscale structure of SHM signals, enabling more accurate capture of both local and global temporal dependencies. To the best of our knowledge, this study introduces the first HAT-based modeling strategy into the domain of SHM anomaly detection.

2. The HAT framework demonstrated high detection accuracy even under extreme label scarcity: Achieving 96.3% accuracy and an 88.3% F1-score with only 20% labeled data, the framework showed strong robustness to limited annotations. This combination of high precision and label efficiency is critical in engineering practice as it delivers dependable early-warning information while sharply reducing annotation labor and total monitoring costs, thus facilitating large-scale deployment in resource-constrained SHM projects.

3. The model enables end-to-end learning from raw time-series inputs and facilitates anomaly visualization through progressive feature separation: This capability eliminates the need for manual feature engineering while generating interpretable representations, thereby enhancing usability and applicability in complex and evolving SHM environments.

This study proposes a scalable and generalizable framework capable of accurately detecting anomalies under real-world constraints, such as sparse labels and complex signal patterns. Its rapid training and minimal reliance on manual feature engineering lower the barrier to long-term deployment. Nevertheless, several limitations remain. (i) Validation is currently restricted to a single bridge dataset; further evaluation on other structures, such as buildings, tunnels, and offshore platforms, is needed to confirm robustness and transferability. (ii) Although high accuracy is achieved with few labels, some annotation is still required. Given the abundance of historical SHM data, future work should exploit these unlabeled records through unsupervised, self-supervised, or semi-supervised learning, thus further reducing labeling effort while preserving detection performance.

## Figures and Tables

**Table 1:** Summary of data categories and descriptions.

Class Name	Number of Samples	Description
normal	13,575	The time response is symmetrical.
missing	2942	The time-domain response is missing.
minor	1775	Compared to normal sensor data, the amplitude in the time domain is very small.
outlier	527	One or more outliers are significantly larger than the normal values.
square	2996	The time response in the time domain is similar to a square wave.
trend	5778	The data have an obvious trend item in the time domain.
drift	679	The vibration response is nonstationary, with random drift.

**Table 2:** Sample distribution in the full dataset and the 20% training subset.

Class Name	Number of Samples	Number of Samples (20%)
normal	13,575	2762
missing	2942	582
minor	1775	365
outlier	527	103
square	2996	574
trend	5778	1132
drift	679	136

**Table 3:** Hardware and software configurations for the experiments.

Parameters	Configuration
CPU	Intel Xeon Gold 6430
GPU	NVIDIA GeForce RTX 4090
GPU memory size	24 G
Operating systems	Win11
Deep learning architecture	Python 3.8 + PyTorch 2.0.0 + CUDA 11.8

**Table 4:** Key parameters used for model training.

Parameters	Setup
Epoch	25
Learning rate	0.0001
Dropout	0.1
Optimizer	Adam
Loss function	CrossEntropyLoss
$D_{\mathrm{model}}$	64
Dim_feedforward	256
Nhead	4

**Table 5:** Performance comparison of different models under varying labeled data proportions.

	HAT			1D-CNN			LSTM			RNN		
	Case 1	Case 2	Case 3	Case 1	Case 2	Case 3	Case 1	Case 2	Case 3	Case 1	Case 2	Case 3
Prediction	90.26	91.19	92.79	85.29	85.67	88.09	67.99	70.41	72.04	70.04	69.90	71.21
Recall	86.47	86.59	87.63	73.50	79.31	79.83	62.63	64.77	67.31	61.07	63.17	65.94
Accuracy	96.28	95.51	96.23	93.27	94.36	94.72	87.85	89.23	90.13	88.76	89.59	90.17
F1	88.32	88.83	90.14	78.96	82.37	83.66	65.20	67.47	69.59	65.25	66.36	68.47

**Table 6:** Performance comparison of HAT against Bao et al. [38] and Tang et al. [18].

	HAT	Bao et al.	Tang et al.
Prediction	90.26	79.16	89.19
Recall	86.47	80.71	79.47
Accuracy	96.28	87.00	94.20
F1	88.32	79.93	84.05

**Table 7:** Resource demands and inference time for different models.

	HAT	1D-CNN	LSTM	RNN
Parameters	200,519	42,663	376,583	100,871
Model Size	0.76 MB	0.16 MB	1.44 MB	0.38 MB
Inference Time	78.81 ms	1.72 ms	3.83 ms	1.54 ms
GPU Memory	9.33 MB	8.43 MB	9.70 MB	8.65 MB

## Data Availability

The data presented in this study are available on request from the corresponding author. The dataset was originally publicly available during the IPC-SHM 2020 competition but has since been restricted.
